# Clinical and Radiological Results of Oxford Phase-3 Medial Unicompartmental Knee Arthroplasty

**DOI:** 10.7759/cureus.6070

**Published:** 2019-11-04

**Authors:** Sinan Karaca, Mehmet N Erdem, Ahmet Oztermeli, Emre Bal, Abdullah Gogus, Azmi Hamzaoglu

**Affiliations:** 1 Orthopaedics and Traumatology, Sehit Prof. Dr. Ilhan Varank Sancaktepe Education and Research Hospital, Istanbul, TUR; 2 Orthopaedics and Traumatology, Isik University, Istanbul, TUR; 3 Orthopaedics and Traumatology, Gebze Fatih Government Hospital, Izmit, TUR; 4 Orthopaedics and Traumatology, Uskudar Goverment Hospital, Istanbul, TUR; 5 Orthopaedics and Traumatology, Istanbul Florence Nightingale Hospital, Istanbul, TUR

**Keywords:** knee, unicompartmental knee arthroplasty, osteoarthritis of knee, arthroplasty

## Abstract

Purpose

The aim of this retrospective study was to investigate the effectiveness of medial unicompartmental knee arthroplasty (UKA) by showing the results of the radiological and clinical outcomes of the patients.

Materials and methods

Seventy-two knees of 54 patients who underwent UKA between September 2005 and March 2011 for medial knee arthritis with a minimum follow-up of six months were evaluated. Range of motion (ROM), Hospital for Special Surgery (HSS) knee score, Knee Society Score (KSS), and Oxford Knee Score (OKS) were investigated both preoperatively and postoperatively. On the other hand, Oxford radiographic evaluation criteria were used to evaluate prostheses radiologically at the final follow-up.

Results

The average age was 53.4 years (47 to 79 years). The average follow-up time was 39.8 months (8 to 72 months). There was a significant difference between preoperative and postoperative ROM, HSS, and OKS (p<0.05). Radiologically, there was no sign of arthritis on the unoperated side of the knee or failure of prosthesis detected. Before the operation, the average clinical KSS was 63.2 and improved to 91.4 after the operation. In addition, the average functional KSS was 54.9 before the operation and improved to 86.5 after the operation. The average knee flexion degree was 109.1 before the operation and there was an improvement to 123.6 degrees after the operation. Before the operation, the average HSS score was 67.5 (range, 52 to 75) and improved to 89.9 (range, 85 to 100) at the final control examination.

Conclusion

This study supports the use of Oxford Phase 3 UKA, which has excellent clinical and radiological results in patients with medial knee arthritis.

## Introduction

The incidence of symptomatic knee osteoarthritis is currently increasing all over the world with the aging of the population [1-3]. The treatment options of this patient population with knee osteoarthritis are debatable. Only limited pain relief and functional improvement can be achieved with the non-operative treatment options [4]. Due to this reason, arthroplasty surgery at the knee is assumed to be popular and by 2030, there will be a linear increase at the rate of 673% [5]. Unicompartmental knee arthroplasty (UKA), high tibial osteotomy (HTO), and conventional total knee arthroplasty (TKA) are the primary surgical methods of treating isolated lateral or anteromedial compartment knee arthritis [6]. OA has a multifactorial etiology, which occurs due to an interplay between systemic and local factors. Osteoarthritis is a disease that can be seen especially in the elderly population. The etiology of osteoarthritis is linked to several responsible genes. Trauma at the knee, high body mass index (BMI), family history, age, diabetes, synovitis, systemic inflammatory mediators, lower limb alignment, joint shape and dysplasia, and inflammatory diseases are causes of osteoarthritis. There is a 3.86 times increased risk of OA at a knee joint with a previous injury [7]. Surgical techniques and instruments are improving currently. There are some studies showing excellent results after UKA, including a decreased angle of deformity, reduced knee pain, and achieving the physiological knee motion range. In addition, these studies have shown that clinical and functional scores increased significantly [8-9].

Shorter incisions and decreased soft tissue damage are the advantages of UKA and there is more bone stock conserved in UKA than in TKA by providing better kinesiology and faster recovery to the patients [10-12].

The aim of this study was to investigate the clinical and radiological results of Oxford medial UKA due to medial compartment arthrosis.

## Materials and methods

This is a retrospective investigation of patients who underwent UKA. After the operation, there was a routine control physical and radiological examination. The first control was in the sixth week. The second was in the sixth month. After these controls, in the first and second-year, the final follow-up control was done. Between September 2005 and March 2011, the 72 consecutive knees of 54 patients who had a UKA operation (Oxford Partial Knee, Biomet Orthopedics, Warsaw, Indiana), with no loss to follow-up, were enrolled in the study. Every participant signed an informed consent form.

Clinically, knee pain resistant to conservative therapy on the anteromedial side was the indication for the operation. On the other hand, radiologically, grade 4 arthritis was the indication for the operation. The exclusion criteria were joint inflammation, ruptured anterior cruciate ligament, and a history of knee surgery. Age and weight were not considered exclusion criteria.

**Figure 1:**
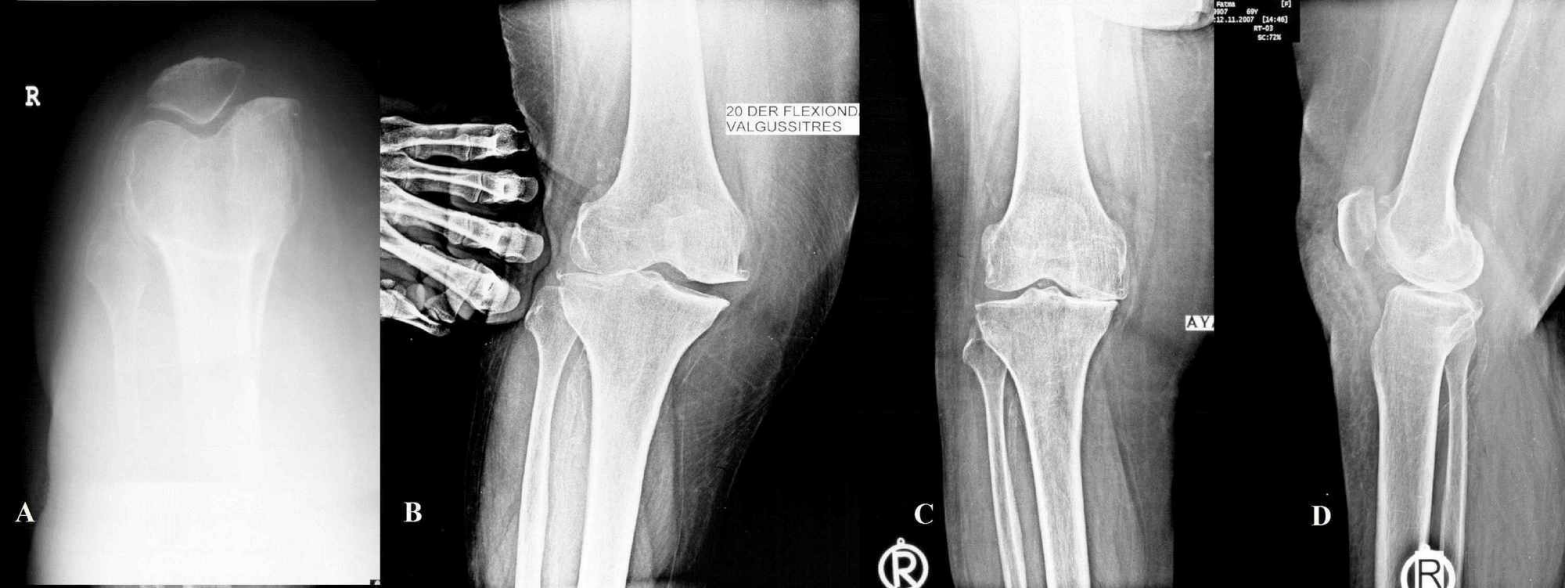
Preoperative radiological images A. Patella tangential, B. Valgus stress, C. AP standing upright, D. Lateral at 20° of flexion views

All patients had a routine physical examination. Also, all patients had a radiological examination, including anteroposterior (AP) standing upright, patella tangential, lateral at 20° of flexion, and varus/valgus stress views of both knees, which were the routine radiograph imaging (Figure 1).

Before the operation, the knee range of motion and knee scores are taken from all patients. At the follow-up examinations, knee scores and range of motion were analyzed and noted from all patients. Implants were assessed with the Oxford radiologic evaluation criteria.

There was no ligament injury, including anterior and posterior cruciate ligaments, in any patient. At the physical examination, if an injury to the ligament is suspected, magnetic resonance imaging (MRI) was done. In some cases before the knee arthroplasty operation, a diagnostic knee arthroscopy operation was done if needed. All the patients were operated by the same senior author (AG) at Bilim University's orthopedic department between the years 2005 and 2011. In all operations, the same implant was used (Oxford Phase III).

Full weight-bearing was allowed immediately postoperatively. Intravenous first-generation cephalosporin (single-shot 2 g cefazolin) was administered preoperatively one hour before surgery. For thrombosis, prophylaxis anticoagulation therapy consisted of low molecular weight heparin (Fundoparinux 2500 IU,) which was prescribed for 21 days.

Statistical analysis was achieved using Statistical Package for the Social Sciences (SPSS) version 22 (IBM Corp, Armonk, New York). Data were studied by using a paired t-test before and after the operation. A p-value of <0.05 was considered statistically significant.

## Results

Changes in knee scores during the follow-up period in patients. At last follow-up control, we had two cases exitus. Exitus was not related to the UKA procedure. The rest of the knees (n=72) were examined at an average follow-up time of 39.8 months. The sexual orientation proportion was 47 females/seven males. Individually, 11 patients were in the 46-55 age group, 25 patients were in the 56-65 age group, and 18 patients were in the 66-79 age group. The mean age was 53.4 (47-79). The average BMI was 26.78 kg/m^2^ (22.47-31.74 kg/m^2^) (Table 1 ).

**Table 1:** Demography of the patient population

DEMOGRAPHIC	
N	54
Male/Female	7/47
Age	53,4
BMI	26.78

All patients had a full range of motion both actively and passively on the seventh day and in the second month after the operation.

Before the operation, the average KSS was 55.11 (range, 43-68), and it increased to 96.71 (range, 88-100) after the operation. Before the operation, the functional KSS was 52.76 (range, 41-67) and it improved to 89.62 (range, 81-99) (p<0.05). Intercalarily, the HSS score improved from 53.21 (range, 38-69) to 95,74 (range, 86-100) (p<0.05). Before the operation, the average ROM was 120.38 degrees (range, 102-130), and it rose to 129.02 degrees (range, 115-140) after the UKA operation (p<0.05). Before the operation, the average Oxford Knee Score was 14.18 (range, 5-22) and it rose to 43.64 (range, 38-48) after the UKA operation (p<0.05) (Table 2).

**Table 2:** Results of the knee scores at the patients who had UKA surgery

	Preoperative	Postoperative	P values
Oxford knee score	14.18±4,7	43,64±5,0	0.024
HSS knee score	53.21±9,5	95,74±5,3*	0.038
KSS clinical knee score	55.11±8,7	96.71±4,3	<0.001
KSS functional knee score	52.76±8,7	89.62±5,1	<0.001
Knee ROM	120,38±5,2	129,02±4,8	0.046

Bone cracks around the implant, disengagement, any infectious disease, or any complications were not seen during the subsequent period.

The weight-bearing radiograph was taken preoperatively and at the last follow-up. The mean femorotibial angle evaluated, and it was 2.8° varus (range, 8.7° varus to 2.1° valgus) before the operation and 2.8° valgus (range, 2.4° varus to 7.4° valgus) after the operation at the final examination. Osteolysis and ≥2 mm abnormal radiolucency around the femoral or tibial part were not observed in any patients on radiographs.

Despite the fact that patellofemoral joint inflammation was suspected in two patients, no treatment was done. Dynamic horizontal compartment joint pain was seen in three patients, however, none of them was treated with TKA.

Even though patellofemoral joint arthritis was suspected in two patients, no treatment was required in light of the fact that they were asymptomatic. Progressive lateral compartment arthritis was observed in three patients but none of them were treated with TKA.

## Discussion

One compartment arthritis is a challenging disease with the absence of high-quality treatment. However, there are some treatment options like TKA, UKA, and HTO. Over the years, the most preferred procedure of orthopedic surgeons was TKA. In a study, TKA and UKA were compared, and it is found that patients with UKA have reduced operation time and greater knee range of motion. In another study, it is shown that the activity levels in patients operated with UKA were better than in patients with TKA [13-15]. Since the patellofemoral compartment is preserved, the failure of the UKA can easily be revised to TKA. A study comparing primary TKA and TKA after failed UKA showed similar clinical results [16]. In our study, we found similar satisfactory results with UKA.

In a study, it is found that the clinical results of HTO and UKA are similar when a convenient patient selection was achieved. [17]. But as compared to HTO, UKA has increasing popularity over the years. There are points of interest like prompt weight-bearing and shorter recovery time after UKA. On the other hand, there are restrictions on weight-bearing and a longer rehabilitation period with HTO. With UKA, this process may be easier for patients, and thus UKA could be preferred. On the other hand, with reduced hospitalization time, rehabilitation period, and return to work time, UKA is more cost-effective than HTO.

In our study, functional recovery was achieved at the one-year follow-up and after that, no significant progression was observed. The literature contains clinical results that are similar to our study [18].

Patient selection is important for achieving good clinical outcomes in UKA surgery. UKA is one of the best choices of surgery for selected patients. However, many complications have been published in studies over the years [19-21].

In a study that shows perfect outcomes, it has been shown that convenient patient selection is the most important factor to achieve successful results [22]. We also use the same criteria for patient selection in our study. Due to this convenient patient selection criteria, we had similar, successful clinical and radiological results that are compatible with the literature.

In orthopedic surgery, less invasive techniques are gaining popularity because of faster recovery time and allowing for a more active lifestyle, therefore, UKA is a good surgical option for the treatment of knee osteoarthritis. Additionally, the Oxford UKA was found to be ideally appropriate for use as an outpatient procedure that lowers costs and has higher patient and surgeon satisfaction.

The main limitations of our present study are a relatively short minimum follow-up, the lack of a control group (e.g. HTO or TKA), and that the data were analyzed retrospectively.

## Conclusions

Oxford mobile-bearing UKA demonstrated excellent clinical improvement and survivorship in patients. Further prospective, randomized follow-up studies should be performed to determine the long-term efficacy of the procedure.
